# *catena*-Poly[[[*N*,*N*′-bis­(3-methoxy­benzyl­idene)ethyl­enediamine]copper(I)]-μ-thio­cyanato-κ^2^ *N*:*S*]

**DOI:** 10.1107/S1600536808041925

**Published:** 2008-12-13

**Authors:** Aliakbar Dehno Khalaji, Hassan Hadadzadeh, Kazuma Gotoh, Hiroyuki Ishida

**Affiliations:** a Department of Science, Gorgan University of Agricultural Sciences and Natural Resources, Gorgan 49189-43464, Iran; b Department of Chemistry, Faculty of Science, Okayama University, Okayama 700-8530, Japan

## Abstract

In the cyrstal structure of the title compound, [Cu(NCS)(C_18_H_20_N_2_O_2_)]_*n*_, the Cu^I^ atom is coordinated in a distorted tetra­hedral geometry by two imino N atoms from a bidentate chelating Schiff base ligand, and one N and one S atoms from two thio­cyanate anions. The thio­cyanate anion bridges the Cu^I^ atoms, forming a zigzag chain along [101]. The Schiff base ligand adopts an *E*,*E* configuration and the dihedral angle between the terminal benzene rings is 53.68 (8)°.

## Related literature

For related copper(I) complexes with bidentate ligands, see: Amirnasr *et al.* (2006 ▶); Khalaji, Brad & Zhang (2008 ▶); Khalaji, Welter *et al.* (2008 ▶); Khalaji & Welter (2006 ▶); Zhao *et al.* (2008 ▶).
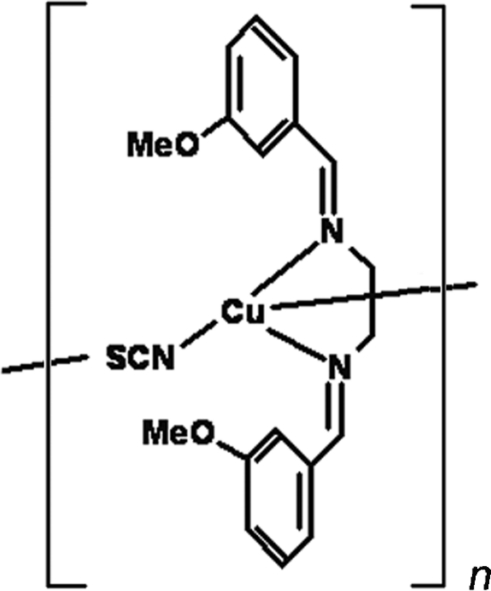


## Experimental

### 

#### Crystal data

[Cu(NCS)(C_18_H_20_N_2_O_2_)]
*M* *_r_* = 417.99
Monoclinic, 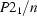

*a* = 8.1316 (3) Å
*b* = 23.5113 (9) Å
*c* = 10.1597 (4) Å
β = 107.1245 (15)°
*V* = 1856.27 (11) Å^3^
*Z* = 4
Mo *K*α radiation
μ = 1.31 mm^−1^
*T* = 193 (1) K
0.31 × 0.17 × 0.02 mm

#### Data collection

Rigaku R-AXIS RAPID diffractometer
Absorption correction: numerical (**ABSCOR**; Higashi, 1995 ▶) *T*_min_ = 0.771, *T*_max_ = 0.974
28362 measured reflections
5395 independent reflections
4614 reflections with *I* > 2σ(*I*)
*R*_int_ = 0.031

#### Refinement

*R*[*F* ^2^ > 2σ(*F* ^2^)] = 0.030
*wR*(*F* ^2^) = 0.076
*S* = 1.05
5395 reflections
235 parameters
H-atom parameters constrained
Δρ_max_ = 0.51 e Å^−3^
Δρ_min_ = −0.23 e Å^−3^

### 

Data collection: *PROCESS-AUTO* (Rigaku/MSC, 2004 ▶); cell refinement: *PROCESS-AUTO*; data reduction: *CrystalStructure* (Rigaku/MSC, 2004 ▶); program(s) used to solve structure: *SHELXS97* (Sheldrick, 2008 ▶); program(s) used to refine structure: *SHELXL97* (Sheldrick, 2008 ▶); molecular graphics: *ORTEP-3* (Farrugia, 1997 ▶); software used to prepare material for publication: *CrystalStructure* and *PLATON* (Spek, 2003 ▶).

## Supplementary Material

Crystal structure: contains datablocks global, I. DOI: 10.1107/S1600536808041925/lh2744sup1.cif

Structure factors: contains datablocks I. DOI: 10.1107/S1600536808041925/lh2744Isup2.hkl

Additional supplementary materials:  crystallographic information; 3D view; checkCIF report

## Figures and Tables

**Table 1:** Selected bond lengths (Å)

Cu1—S1	2.3130 (4)
Cu1—N1^i^	1.9347 (12)
Cu1—N2	2.0917 (12)
Cu1—N3	2.0900 (13)

Symmetry code: (i) 
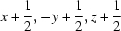
.

**Fig. 1.**
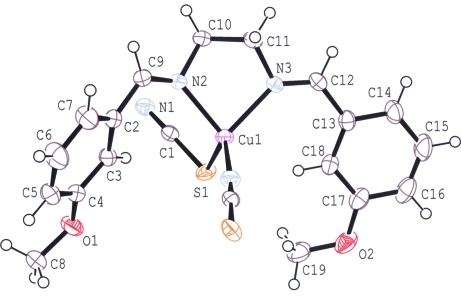
The molecular structure of the title compound with atom-labeling scheme. Displacement ellipsoids are drawn at the 50% probability level.

**Fig. 2.**
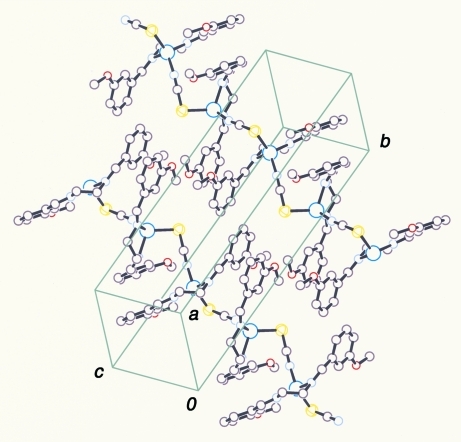
A partial packing view of the title compound, (I).

## supplementary crystallographic information

### Comment

Synthesis and characterization of copper(I) complexes with bidentate chelating Schiff base ligands have received much attention in recent years (Khalaji, Brad & Zhang, 2008; Khalaji, Welter *et al.*, 2008; Zhao *et al.*, 2008). Depending on the ligands involved, copper(I) complexes can show a wide variety of structures (Amirnasr *et al.*, 2006; Khalaji & Welter, 2006; Khalaji, Brad & Zhang, 2008; Khalaji, Welter *et al.*, 2008). As part of a general study of transition metal complexes with bidentate chelating Schiff base ligands (Khalaji & Welter, 2006; Khalaji, Brad & Zhang, 2008; Khalaji, Welter *et al.*, 2008), here, we reported the synthesis and the crystal structure of the title compound, (I).

The crystal structure of the title compound, (I), is shown in Fig. 1. The Schiff base (3-MeO-ba)_2_en ligand chelates the Cu^I^ atom to form a five-membered ring, with N2—Cu1—N3 = 83.78 (4)°, which is in good agreement with the corresponding angles in related complexes (Khalaji & Welter, 2006; Khalaji, Brad & Zhang, 2008; Khalaji, Welter *et al.*, 2008). The Cu—N and Cu—S distances (Table 1) are similar to those in the other copper(I) complexes. The C12—N3 and C9—N2 bond lengths of 1.2717 (18) and 1.2665 (18) Å, respectively, conform to the value for a C=N double bond, while the N2—C10 and N3—C11 bond lengths of 1.462 (2) and 1.476 (2) Å, respectively, conform to the value for a C—N single bond. These C—N lengths are comparable to the corresponding values observed in other tetrahedral copper(I) complexes with bidentate chelating Schiff base ligands (Khalaji & Welter, 2006; Khalaji, Brad & Zhang, 2008; Khalaji, Welter *et al.*, 2008). The bidentate chelating (3-MeO-ba)_2_en ligand adopts an *E*,*E* configuration in this structure.

### Experimental

The title compound, (I), was synthesized using a method analogous to the literature procedure (Khalaji & Welter, 2006), except that CuI was replaced with CuNCS. Single crystals suitable for data collection were obtained by slow evaporation from an acetonitrile solution at 273 K.

### Refinement

H atoms were positioned geometrically (C—H = 0.95–0.99 Å) and treated as riding, with *U*_iso_(H) = 1.2*U*_eq_(C) or 1.5*U*_eq_(methyl C).

### Crystal data

**Table:**

[Cu(NCS)(C_18_H_20_N_2_O_2_)]	*F*(000) = 864.00
*M**_r_* = 417.99	*D*_x_ = 1.496 Mg m^−^^3^
Monoclinic, *P*2_1_/*n*	Mo *K*α radiation, λ = 0.71075 Å
Hall symbol: -P 2yn	Cell parameters from 22727 reflections
*a* = 8.1316 (3) Å	θ = 3.1–30.0°
*b* = 23.5113 (9) Å	µ = 1.31 mm^−^^1^
*c* = 10.1597 (4) Å	*T* = 193 K
β = 107.1245 (15)°	Platelet, yellow
*V* = 1856.27 (11) Å^3^	0.31 × 0.17 × 0.02 mm
*Z* = 4	

### Data collection

**Table:**

Rigaku R-AXIS RAPID diffractometer	4614 reflections with *I* > 2σ(*I*)
Detector resolution: 10.00 pixels mm^-1^	*R*_int_ = 0.031
ω scans	θ_max_ = 30.0°, θ_min_ = 3.1°
Absorption correction: numerical (*ABSCOR*; Higashi, 1995)	*h* = −10→11
*T*_min_ = 0.771, *T*_max_ = 0.974	*k* = −32→32
28362 measured reflections	*l* = −14→12
5395 independent reflections	

### Refinement

**Table:**

Refinement on *F*^2^	Primary atom site location: structure-invariant direct methods
Least-squares matrix: full	Secondary atom site location: difference Fourier map
*R*[*F*^2^ > 2σ(*F*^2^)] = 0.030	Hydrogen site location: inferred from neighbouring sites
*wR*(*F*^2^) = 0.076	H-atom parameters constrained
*S* = 1.05	*w* = 1/[σ^2^(*F*_o_^2^) + (0.0358*P*)^2^ + 0.7637*P*] where *P* = (*F*_o_^2^ + 2*F*_c_^2^)/3
5395 reflections	(Δ/σ)_max_ = 0.001
235 parameters	Δρ_max_ = 0.51 e Å^−^^3^
0 restraints	Δρ_min_ = −0.23 e Å^−^^3^

### Special details

**Table:**

Geometry. All e.s.d.'s (except the e.s.d. in the dihedral angle between two l.s. planes) are estimated using the full covariance matrix. The cell e.s.d.'s are taken into account individually in the estimation of e.s.d.'s in distances, angles and torsion angles; correlations between e.s.d.'s in cell parameters are only used when they are defined by crystal symmetry. An approximate (isotropic) treatment of cell e.s.d.'s is used for estimating e.s.d.'s involving l.s. planes.
Refinement. Refinement of *F*^2^ against ALL reflections. The weighted *R*-factor *wR* and goodness of fit *S* are based on *F*^2^, conventional *R*-factors *R* are based on *F*, with *F* set to zero for negative *F*^2^. The threshold expression of *F*^2^ > σ(*F*^2^) is used only for calculating *R*-factors(gt) *etc*. and is not relevant to the choice of reflections for refinement. *R*-factors based on *F*^2^ are statistically about twice as large as those based on *F*, and *R*- factors based on ALL data will be even larger.

### Fractional atomic coordinates and isotropic or equivalent isotropic displacement parameters (Å^2^)

**Table:**

	*x*	*y*	*z*	*U*_iso_*/*U*_eq_	
Cu1	0.42300 (2)	0.235166 (8)	0.471370 (18)	0.02525 (6)	
S1	0.16168 (5)	0.188164 (18)	0.41646 (4)	0.03209 (9)	
O1	−0.06043 (17)	0.34954 (5)	0.56184 (14)	0.0406 (3)	
O2	0.46009 (18)	0.04893 (6)	0.71225 (15)	0.0472 (3)	
N1	0.01289 (17)	0.24053 (6)	0.16100 (13)	0.0302 (3)	
N2	0.41448 (15)	0.30353 (5)	0.33778 (12)	0.0248 (2)	
N3	0.61360 (15)	0.20470 (5)	0.38943 (12)	0.0245 (2)	
C1	0.07436 (18)	0.21886 (6)	0.26548 (15)	0.0255 (3)	
C2	0.3075 (2)	0.38111 (6)	0.44500 (16)	0.0277 (3)	
C3	0.1706 (2)	0.35321 (6)	0.47169 (16)	0.0281 (3)	
H3	0.1399	0.3160	0.4364	0.034*	
C4	0.0781 (2)	0.37944 (6)	0.55001 (15)	0.0294 (3)	
C5	0.1271 (2)	0.43268 (7)	0.60751 (18)	0.0374 (4)	
H5	0.0657	0.4504	0.6626	0.045*	
C6	0.2674 (3)	0.45947 (8)	0.5830 (2)	0.0476 (5)	
H6	0.3037	0.4954	0.6240	0.057*	
C7	0.3549 (2)	0.43480 (7)	0.5002 (2)	0.0411 (4)	
H7	0.4472	0.4545	0.4809	0.049*	
C8	−0.1738 (2)	0.37722 (8)	0.6249 (2)	0.0428 (4)	
H8A	−0.1147	0.3833	0.7228	0.064*	
H8B	−0.2757	0.3534	0.6151	0.064*	
H8C	−0.2091	0.4140	0.5800	0.064*	
C9	0.39553 (19)	0.35649 (6)	0.35092 (16)	0.0283 (3)	
H9	0.4406	0.3817	0.2971	0.034*	
C10	0.4946 (2)	0.28431 (7)	0.23429 (15)	0.0289 (3)	
H10A	0.5255	0.3175	0.1866	0.035*	
H10B	0.4127	0.2604	0.1649	0.035*	
C11	0.6547 (2)	0.25029 (7)	0.30428 (17)	0.0299 (3)	
H11A	0.7020	0.2334	0.2338	0.036*	
H11B	0.7432	0.2758	0.3631	0.036*	
C12	0.70674 (18)	0.16036 (6)	0.40348 (16)	0.0277 (3)	
H12	0.7894	0.1591	0.3541	0.033*	
C13	0.6977 (2)	0.11098 (6)	0.48926 (16)	0.0291 (3)	
C14	0.8217 (3)	0.06895 (8)	0.5020 (2)	0.0423 (4)	
H14	0.9060	0.0724	0.4547	0.051*	
C15	0.8215 (3)	0.02186 (8)	0.5846 (2)	0.0531 (5)	
H15	0.9069	−0.0067	0.5942	0.064*	
C16	0.6993 (3)	0.01612 (7)	0.6521 (2)	0.0471 (5)	
H16	0.6997	−0.0165	0.7075	0.056*	
C17	0.5745 (2)	0.05804 (7)	0.63967 (18)	0.0359 (3)	
C18	0.5742 (2)	0.10550 (7)	0.55896 (17)	0.0307 (3)	
H18	0.4900	0.1343	0.5511	0.037*	
C19	0.3301 (3)	0.09082 (10)	0.7005 (2)	0.0532 (5)	
H19A	0.3841	0.1279	0.7280	0.080*	
H19B	0.2599	0.0807	0.7606	0.080*	
H19C	0.2569	0.0927	0.6048	0.080*	

### Atomic displacement parameters (Å^2^)

**Table:**

	*U*^11^	*U*^22^	*U*^33^	*U*^12^	*U*^13^	*U*^23^
Cu1	0.02571 (10)	0.02875 (10)	0.02277 (9)	−0.00058 (7)	0.00944 (7)	0.00156 (6)
S1	0.02396 (18)	0.0417 (2)	0.02944 (18)	−0.00463 (15)	0.00609 (14)	0.01091 (15)
O1	0.0427 (7)	0.0376 (6)	0.0521 (7)	−0.0008 (5)	0.0304 (6)	−0.0038 (5)
O2	0.0445 (7)	0.0437 (7)	0.0519 (8)	−0.0052 (6)	0.0118 (6)	0.0206 (6)
N1	0.0276 (6)	0.0381 (7)	0.0254 (6)	−0.0013 (5)	0.0084 (5)	−0.0009 (5)
N2	0.0246 (6)	0.0277 (6)	0.0243 (6)	0.0011 (5)	0.0103 (4)	0.0022 (4)
N3	0.0219 (6)	0.0267 (6)	0.0265 (6)	−0.0017 (4)	0.0097 (4)	−0.0012 (5)
C1	0.0207 (6)	0.0308 (7)	0.0267 (7)	−0.0019 (5)	0.0094 (5)	−0.0021 (5)
C2	0.0309 (7)	0.0231 (6)	0.0304 (7)	0.0032 (5)	0.0109 (6)	0.0021 (5)
C3	0.0325 (8)	0.0231 (6)	0.0310 (7)	0.0022 (5)	0.0127 (6)	−0.0001 (5)
C4	0.0332 (8)	0.0288 (7)	0.0286 (7)	0.0039 (6)	0.0127 (6)	0.0026 (6)
C5	0.0460 (10)	0.0326 (8)	0.0382 (9)	0.0058 (7)	0.0193 (7)	−0.0056 (7)
C6	0.0555 (12)	0.0295 (8)	0.0619 (12)	−0.0046 (8)	0.0238 (10)	−0.0167 (8)
C7	0.0429 (10)	0.0291 (8)	0.0558 (11)	−0.0066 (7)	0.0218 (8)	−0.0073 (7)
C8	0.0414 (10)	0.0485 (10)	0.0466 (10)	0.0115 (8)	0.0253 (8)	0.0059 (8)
C9	0.0289 (7)	0.0272 (7)	0.0308 (7)	0.0003 (5)	0.0122 (6)	0.0047 (6)
C10	0.0337 (8)	0.0311 (7)	0.0262 (7)	0.0027 (6)	0.0156 (6)	0.0025 (6)
C11	0.0282 (7)	0.0314 (7)	0.0349 (8)	−0.0005 (6)	0.0170 (6)	0.0022 (6)
C12	0.0218 (7)	0.0315 (7)	0.0310 (7)	−0.0002 (5)	0.0096 (5)	−0.0033 (6)
C13	0.0280 (7)	0.0268 (7)	0.0300 (7)	0.0022 (6)	0.0048 (6)	−0.0038 (6)
C14	0.0468 (10)	0.0380 (9)	0.0434 (10)	0.0151 (8)	0.0154 (8)	−0.0034 (7)
C15	0.0703 (14)	0.0342 (9)	0.0539 (11)	0.0251 (9)	0.0167 (10)	0.0000 (8)
C16	0.0649 (13)	0.0244 (7)	0.0448 (10)	0.0057 (8)	0.0052 (9)	0.0040 (7)
C17	0.0369 (9)	0.0293 (7)	0.0363 (8)	−0.0051 (6)	0.0026 (7)	0.0032 (6)
C18	0.0270 (7)	0.0266 (7)	0.0358 (8)	0.0002 (6)	0.0050 (6)	0.0030 (6)
C19	0.0392 (10)	0.0670 (13)	0.0561 (12)	−0.0005 (9)	0.0183 (9)	0.0273 (10)

### Geometric parameters (Å, °)

**Table:**

Cu1—S1	2.3130 (4)	C14—C15	1.389 (2)
Cu1—N1^i^	1.9347 (12)	C15—C16	1.370 (3)
Cu1—N2	2.0917 (12)	C16—C17	1.394 (2)
Cu1—N3	2.0900 (13)	C17—C18	1.384 (2)
S1—C1	1.6542 (14)	C3—H3	0.950
O1—C4	1.363 (2)	C5—H5	0.950
O1—C8	1.425 (2)	C6—H6	0.950
O2—C17	1.364 (2)	C7—H7	0.950
O2—C19	1.424 (2)	C8—H8A	0.980
N1—C1	1.1505 (18)	C8—H8B	0.980
N2—C9	1.2665 (18)	C8—H8C	0.980
N2—C10	1.462 (2)	C9—H9	0.950
N3—C11	1.476 (2)	C10—H10A	0.990
N3—C12	1.2717 (18)	C10—H10B	0.990
C2—C3	1.386 (2)	C11—H11A	0.990
C2—C7	1.389 (2)	C11—H11B	0.990
C2—C9	1.471 (2)	C12—H12	0.950
C3—C4	1.390 (2)	C14—H14	0.950
C4—C5	1.389 (2)	C15—H15	0.950
C5—C6	1.388 (3)	C16—H16	0.950
C6—C7	1.380 (3)	C18—H18	0.950
C10—C11	1.515 (2)	C19—H19A	0.980
C12—C13	1.467 (2)	C19—H19B	0.980
C13—C14	1.390 (2)	C19—H19C	0.980
C13—C18	1.394 (2)		
			
S1—Cu1—N1^i^	115.61 (4)	C4—C3—H3	119.9
S1—Cu1—N2	110.98 (3)	C4—C5—H5	120.6
S1—Cu1—N3	118.46 (3)	C6—C5—H5	120.6
N1^i^—Cu1—N2	110.48 (5)	C5—C6—H6	119.4
N1^i^—Cu1—N3	113.01 (5)	C7—C6—H6	119.4
N2—Cu1—N3	83.78 (4)	C2—C7—H7	120.1
Cu1—S1—C1	97.37 (5)	C6—C7—H7	120.1
C4—O1—C8	117.71 (13)	O1—C8—H8A	109.5
C17—O2—C19	117.00 (15)	O1—C8—H8B	109.5
Cu1^ii^—N1—C1	169.62 (13)	O1—C8—H8C	109.5
Cu1—N2—C9	131.98 (11)	H8A—C8—H8B	109.5
Cu1—N2—C10	107.01 (8)	H8A—C8—H8C	109.5
C9—N2—C10	118.26 (14)	H8B—C8—H8C	109.5
Cu1—N3—C11	107.88 (9)	N2—C9—H9	118.2
Cu1—N3—C12	136.32 (11)	C2—C9—H9	118.2
C11—N3—C12	115.52 (14)	N2—C10—H10A	109.8
S1—C1—N1	179.41 (15)	N2—C10—H10B	109.8
C3—C2—C7	119.65 (17)	C11—C10—H10A	109.8
C3—C2—C9	120.89 (13)	C11—C10—H10B	109.8
C7—C2—C9	119.31 (16)	H10A—C10—H10B	108.3
C2—C3—C4	120.22 (13)	N3—C11—H11A	109.6
O1—C4—C3	114.99 (12)	N3—C11—H11B	109.6
O1—C4—C5	124.75 (16)	C10—C11—H11A	109.6
C3—C4—C5	120.26 (16)	C10—C11—H11B	109.6
C4—C5—C6	118.86 (18)	H11A—C11—H11B	108.1
C5—C6—C7	121.14 (17)	N3—C12—H12	117.2
C2—C7—C6	119.77 (18)	C13—C12—H12	117.2
N2—C9—C2	123.57 (15)	C13—C14—H14	120.2
N2—C10—C11	109.21 (12)	C15—C14—H14	120.2
N3—C11—C10	110.28 (13)	C14—C15—H15	119.7
N3—C12—C13	125.68 (15)	C16—C15—H15	119.6
C12—C13—C14	117.15 (16)	C15—C16—H16	120.0
C12—C13—C18	123.00 (14)	C17—C16—H16	120.0
C14—C13—C18	119.84 (15)	C13—C18—H18	120.0
C13—C14—C15	119.6 (2)	C17—C18—H18	120.0
C14—C15—C16	120.70 (19)	O2—C19—H19A	109.5
C15—C16—C17	120.06 (17)	O2—C19—H19B	109.5
O2—C17—C16	115.76 (16)	O2—C19—H19C	109.5
O2—C17—C18	124.37 (15)	H19A—C19—H19B	109.5
C16—C17—C18	119.87 (18)	H19A—C19—H19C	109.5
C13—C18—C17	119.96 (15)	H19B—C19—H19C	109.5
C2—C3—H3	119.9		
			
S1—Cu1—N1^i^—C1^i^	−141.8 (7)	C11—N3—C12—C13	176.20 (12)
N1^i^—Cu1—S1—C1	138.73 (7)	C12—N3—C11—C10	150.57 (12)
S1—Cu1—N2—C9	100.29 (12)	C3—C2—C7—C6	1.2 (2)
S1—Cu1—N2—C10	−99.44 (8)	C7—C2—C3—C4	1.8 (2)
N2—Cu1—S1—C1	11.92 (7)	C3—C2—C9—N2	−33.1 (2)
S1—Cu1—N3—C11	119.32 (7)	C9—C2—C3—C4	−173.74 (12)
S1—Cu1—N3—C12	−67.25 (13)	C7—C2—C9—N2	151.34 (14)
N3—Cu1—S1—C1	−82.44 (6)	C9—C2—C7—C6	176.78 (15)
N1^i^—Cu1—N2—C9	−29.30 (14)	C2—C3—C4—O1	175.86 (12)
N1^i^—Cu1—N2—C10	130.97 (8)	C2—C3—C4—C5	−3.0 (2)
N2—Cu1—N1^i^—C1^i^	−14.7 (7)	O1—C4—C5—C6	−177.52 (14)
N1^i^—Cu1—N3—C11	−100.84 (9)	C3—C4—C5—C6	1.3 (2)
N1^i^—Cu1—N3—C12	72.59 (14)	C4—C5—C6—C7	1.7 (2)
N3—Cu1—N1^i^—C1^i^	77.2 (7)	C5—C6—C7—C2	−3.0 (2)
N2—Cu1—N3—C11	8.80 (8)	N2—C10—C11—N3	52.70 (16)
N2—Cu1—N3—C12	−177.78 (13)	N3—C12—C13—C14	−173.84 (14)
N3—Cu1—N2—C9	−141.57 (13)	N3—C12—C13—C18	4.8 (2)
N3—Cu1—N2—C10	18.70 (8)	C12—C13—C14—C15	178.56 (15)
C8—O1—C4—C3	−171.75 (13)	C12—C13—C18—C17	−179.16 (13)
C8—O1—C4—C5	7.1 (2)	C14—C13—C18—C17	−0.6 (2)
C19—O2—C17—C16	−179.45 (15)	C18—C13—C14—C15	−0.1 (2)
C19—O2—C17—C18	1.5 (2)	C13—C14—C15—C16	0.7 (2)
Cu1—N2—C9—C2	−25.4 (2)	C14—C15—C16—C17	−0.7 (2)
Cu1—N2—C10—C11	−42.47 (13)	C15—C16—C17—O2	−179.10 (15)
C9—N2—C10—C11	120.98 (14)	C15—C16—C17—C18	−0.0 (2)
C10—N2—C9—C2	176.11 (11)	O2—C17—C18—C13	179.63 (14)
Cu1—N3—C11—C10	−34.46 (13)	C16—C17—C18—C13	0.6 (2)
Cu1—N3—C12—C13	3.1 (2)		

Symmetry codes: (i) *x*+1/2, −*y*+1/2, *z*+1/2; (ii) *x*−1/2, −*y*+1/2, *z*−1/2.
